# Overcoming functional redundancy in three actin depolymerizing factor genes for rice pollen tube growth

**DOI:** 10.1093/plphys/kiae158

**Published:** 2024-03-13

**Authors:** Eui-Jung Kim, Woo-Jong Hong, Minseo Kang, Jong-Seong Jeon, Yu-Jin Kim, Ki-Hong Jung

**Affiliations:** Graduate School of Green-Bio Science and Crop Biotech Institute, Kyung Hee University, Yongin 17104, Republic of Korea; Research Center for Plant Plasticity, Seoul National University, Seoul 08826, Republic of Korea; Department of Smart Farm Science, Kyung Hee University, Yongin 17104, Republic of Korea; Graduate School of Green-Bio Science and Crop Biotech Institute, Kyung Hee University, Yongin 17104, Republic of Korea; Research Center for Plant Plasticity, Seoul National University, Seoul 08826, Republic of Korea; Graduate School of Green-Bio Science and Crop Biotech Institute, Kyung Hee University, Yongin 17104, Republic of Korea; Department of Life Science and Environmental Biochemistry, Life and Industry Convergence Research Institute, Pusan National University, Miryang 50463, Republic of Korea; Graduate School of Green-Bio Science and Crop Biotech Institute, Kyung Hee University, Yongin 17104, Republic of Korea; Research Center for Plant Plasticity, Seoul National University, Seoul 08826, Republic of Korea

Dear Editor,

Functional redundancy caused by genome duplication is widespread in angiosperms undergoing genome duplication early in their evolution, and genes preferentially expressed in the anthers/pollen of rice (*Oryza sativa* L.) have greater potential for functional redundancy than those in other tissues (Kafri et al. 2009; Hong et al. 2020). Effective methods are needed to overcome the functional redundancy of pollen-preferred genes. Unlike Arabidopsis, rice has only 1 ovule in each flower, making it difficult to study the functional roles of late pollen-preferred genes through loss-of-function mutant analysis. Moreover, generating multiple knockout mutants by crossing pollen germination or pollen tube (PT) growth-related mutants is challenging in rice. This challenge can be resolved by predicting functional redundancy and applying CRISPR-Cas9 to multiple targets (Xie et al. 2015; Hong et al. 2020). In this study, we generated homozygous multiple loss-of-function mutants using the CRISPR-Cas9 system to understand the functions of predominant and redundant genes involved in rice male gamete transfer.

Actin depolymerizing factor (ADF) depolymerizes filamentous actin (Jiang et al. 2022). Eleven *ADFs* exist in rice, 9 of which possess the cofilin/tropomyosin-type actin-binding protein domain (Feng et al. 2006). Significant sequence similarity among *OsADF1*, *OsADF6*, and *OsADF9* as well as high Pearson correlation coefficients (PCCs) for expression patterns suggests functional redundancy among the 3 genes (Fig. 1A; Supplementary Figs. S1 and S2; Supplementary Table S1). Meta-expression data were validated by reverse transcription quantitative PCR (RT-qPCR) using 9 rice tissues (Fig. 1B; Supplementary Tables S2 and S3). The 3 OsADFs had a similar subcellular localization in pollen grains (PGs) and PTs, and these signals were detected throughout the entire PTs (Fig. 1C). To investigate their function, single and multiple loss-of-function mutants were generated using CRISPR-Cas9 to simultaneously target these 3 OsADFs (Fig. 1D; Supplementary Fig. S3A; Supplementary Table S3). Reproductive tissue development and the Iodine potassium iodide solution (I_2_-KI) staining pattern of PGs were normal in all mutants (Fig. 1E; Supplementary Fig. S3B). However, the *adf1/6/9* triple mutant displayed nearly complete sterility in 3 independent lines at rates of 0.004% to 0.168% (Fig. 1, F and G; Appendix S1). The reciprocal crossing of *adf1/6/9* with the wild-type Dongjin (DJ) cultivar led to male sterility caused by defects in the male gamete gene transfer process (Supplementary Fig. S4). Single-gene mutants of *adf1*, *adf6*, and *adf9* exhibited fertility rates of 71.76%, 57.60% to 74.42%, and 29.07% to 43.99%, respectively. Regarding double mutants, *adf1/6*, *adf1/9*, and *adf6/9* exhibited fertility rates of 50.3%, 17.31% to 23.70%, and 8.17% to 13.56%, respectively. The *adf1/6/9* defect was also identified in the germination medium, in which 60% of the pollen failed to hydrate and the remaining pollen ruptured before PT elongation (Fig. 1, I and K). We obtained a significant proportion of normal PTs in *adf1/6/9* via treatment with 2 nM latrunculin B (LatB), implying that the germination defect of *adf1/6/9* is attributable to abnormalities in actin filament formation (Jiang et al. 2022; Fig. 1, I and N). During pollen germination in vivo, most PTs stopped in the middle of the stigma in *adf1/6/9*. Even if the PT approaches the ovary, it does not sufficiently elongate for successful fertilization (Fig. 1, E and M). Among double mutants, *adf1/9* and *adf6/9* had low germination rates in the medium. The PT length was the most severely reduced in *adf6/9* in vivo (Fig. 1, H, K, and M). The single-gene mutants exhibited wavy and long PTs in the medium. However, the eventual decrease in fertility rates suggests the instability of wavy PTs within the ovule (Supplementary Fig. S3B). In addition, *adf9*, *adf1/9*, and *adf6/9* had significantly reduced germination and fertility rates compared to those of *adf1*, *adf6*, and *adf1/6*. These results indicated the involvement of these 3 genes in PT growth, and *OsADF9* has a more significant role in pollen germination than the other genes (Fig. 2F).

**Figure 1.**
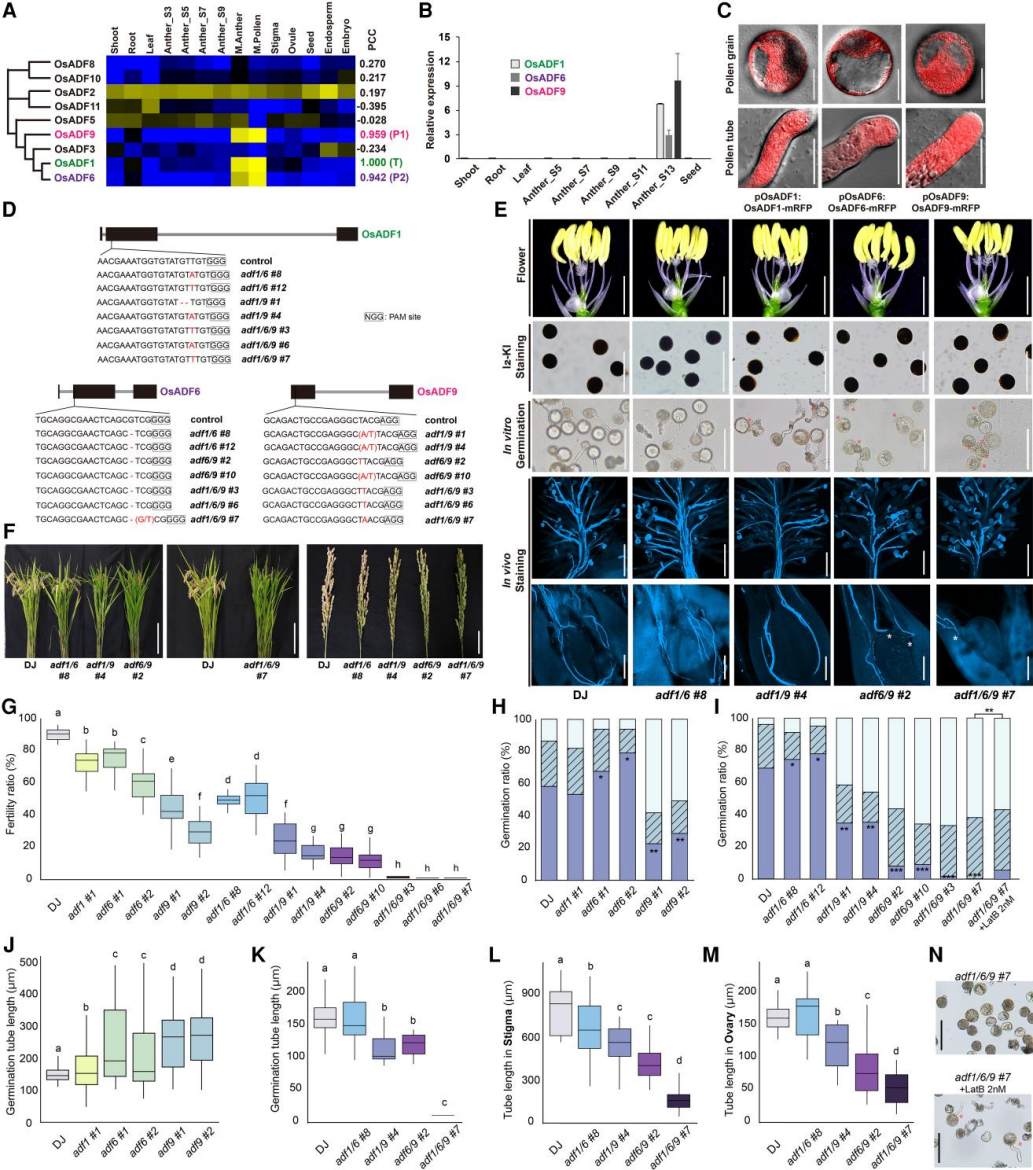
Mutants of 3 *OsADF* genes exhibited gradual decreases in fertility in proportion to the number of mutated genes. **A)** Phylogenetic tree and expression patterns of 9 *OsADF*s. T, a target and queried gene for calculating PCC; P, paralog gene; S, stage number for anther development; M, mature stage. **B)** RT-qPCR of *OsADF1/6/9*. **C)** Subcellular localization in the rice PGs and PTs of OsADF1/6/9. p, promoter; Bar, 20 (PG) or 10 *μ*m (PT). **D)** Edited sequences of multiple mutants. The boxes, lines, and squares indicate exons, introns, and protospacer adjacent motif sites, respectively. **E)** Pictures of PTs were used for in vitro and in vivo germination analyses to compare DJ and a series of multiple mutants. Bar, 100 (in vitro) or 200 *μ*m (in vivo). **F)** Plant and panicle pictures of DJ and mutants. Bar, 30 (plant) or 5 cm (panicle). **G)** Fertility ratio of DJ and mutants. **H and I)** Germination ratio of DJ and single mutants **H)**, multiple mutants, and LatB treatment **I)**. Each bar represents the pollen germination status. Dark bars indicate intact PTs, bars with dashes indicate pollen that can hydrate but not elongate PTs, and light bars represent pollen unable to hydrate. **J and K)** In vitro tube length of DJ and single mutants **J)** and multiple mutants **K)** in medium. **L and M)** Tube length at the stigma in DJ and multiple mutants **L)** and tube length at the ovary **M)**. **N)** LatB treatment of *adf1/6/9* pollen. Bar, 100 *μ*m. *P*-values were calculated using Duncan's multiple-range test with lowercase letters a-h (*P* < 0.05) and 1-way ANOVA with asterisks. *P < 0.05; **P < 0.01; and ***P < 0.001.

**Figure 2.**
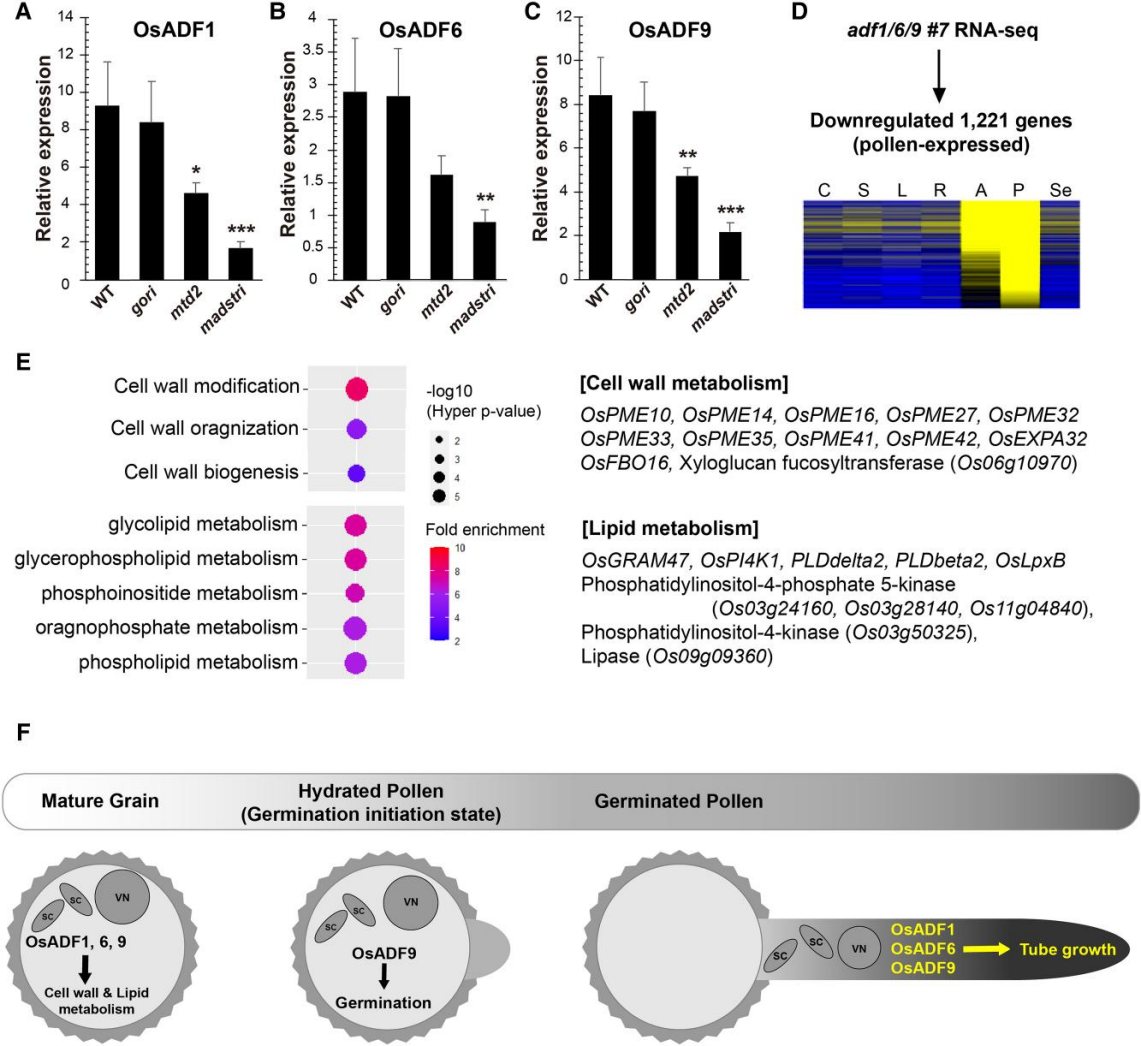
Possible function of *OsADF1/6/9* determined by transcriptome analysis. **A to C)** Expression patterns of *OsADF1*, *OsADF6*, and *OsADF9* in mutant lines such as *osmtd2*, *gori*, and *osmadstri*. **D and E)** The heatmap and GO analysis graph for pollen-expressed and pollen-downregulated genes in *adf1/6/9*. **F)** Functional model of *OsADF1*, *OsADF6*, and *OsADF9*. These 3 genes have functional redundancy, and transcriptome analysis of the triple-mutant anthers indicated that these 3 OsADFs affect cell wall and lipid metabolism. In all graphs, error bars represent the Sd, and *P*-values were calculated by 1-way ANOVA and the Scheffé method. **P < 0.01 and ***P < 0.001.

Transcriptome analysis of previously characterized rice pollen mutants provides clues to the molecular function of *OsADF1/6/9*. The expression of *OsADF*s was analyzed by RT-qPCR in 3 reported mutants, *osmadstri*, *osmtd2*, and *gori* (Kim et al. 2021a, 2021b, 2021c). We found that OsADF1/6/9 was significantly downregulated in *osmtd2* and *osmadstri*, but not in *gori* (Fig. 2, A to C). However, because of the absence of a CArG motif in the 2-kb promoter regions of *OsADF1/6/9*, it is possible that other intermediates link these OsADFs with OsMADS62/63/68. Transcriptome analysis of *adf1/6/9* mature anthers suggests the probable downstream pathways of *OsADF1/6/9*. Among the downregulated genes in *adf1/6/9*, 1,221 genes were significantly expressed in DJ pollen, and their functions were closely associated with cell wall and lipid metabolism (Fig. 2, D and E; Supplementary Table S2; Appendix S1).

Because of the structural characteristics of monocotyledons, such as having a single ovule in the ovary, there are practical limitations to producing homozygous seeds of mutants with pollen germination or PT growth defects. In addition, creating multiple mutants through crossbreeding can be more challenging because of their inherent structural features. We believe that generating multiple knockout mutants using the CRISPR-Cas9 system is a strategy for rapidly creating new male sterile lines and accelerating the study of genes with functional redundancy. This will increase our knowledge of the functional networks of male gamete transfer processes in crops.

## Supplementary Material

kiae158_Supplementary_Data

## Data Availability

The raw data files for the RNA-seq analysis can be found at the GenBank ArrayExpress under the accession number E-MTAB-13557.

## Supplementary data

The following materials are available in the online version of this article.

**Supplementary Appendix S1.** Supplementary description.

**Supplementary Figure S1.** Phylogenetic tree based on protein sequence similarities among OsADFs and ADFs in other species.

**Supplementary Figure S2.** Overview of multiple sequence alignment of proteins according to subgroup based on phylogenetic tree analysis.

**Supplementary Figure S3.** Target site of single mutants for each of *OsADF1*, *OsADF6*, and *OsADF9* by CRISPR-Cas9 gene editing system and the images of pollen germination, PT growth, and whole plant in the single mutants.

**Supplementary Figure S4.** Reciprocal crossing analysis between *adf1/6/9* ovaries and wild-type (DJ) pollen.

**Supplementary Table S1.** Gene locus IDs and amino acid sequences used in phylogenetic analysis.

**Supplementary Table S2.** Genes significantly downregulated in *adf1/6/9*.

**Supplementary Table S3**. List of primers used in this study.

## Dive Curated Terms

The following phenotypic, genotypic, and functional terms are of significance to the work described in this paper:

OsADF9 Gramene: LOC_Os07g30090
OsADF9 Araport: LOC_Os07g30090

OsADF1 Gramene: LOC_Os02g44470
OsADF1 Araport: LOC_Os02g44470

OsADF6 Gramene: LOC_Os04g46910
OsADF6 Araport: LOC_Os04g46910
