# Supplementary material for: Overcoming functional redundancy in three actin depolymerizing factor genes for rice pollen tube growth
Source: Plant Physiol. 2024 Mar 13;195(2):1134–7. doi: 10.1093/plphys/kiae158 (PMC11142328; doi:10.1093/plphys/kiae158)
Supplement: kiae158_Supplementary_Data [file kiae158_supplementary_data.zip › Appendix S1_Revision V2_Final.docx]

**Appendix S1. Supplementary description**

**Sequence alignment and Phylogenetic tree construction.**

Protein sequences were collected from the Rice Genome Annotation Project (RGAP) website (Kawahara et al., 2013) for rice ADFs, and from the National Center for Biotechnology Information (NCBI) for other species. Cluster W from Molecular Evolutionary Genetics Analysis (MEGA-X; Kumar et al., 2018) was used for sequence alignment to generate the phylogenetic tree in Supplemental Figure 1. For paired alignments, the gap opening and extension penalty were set to 10.00 and 0.10, and for multiple alignments, they were set to 10.00 and 0.2. The phylogenetic tree was created using the Neighbor-joining statistical method of MEGA-X, and the bootstrap method replication number of phylogenies was set to 1000. The substitution model method was set to the Poisson model. Multiple sequence alignments and conservation ratios for each subgroup in Supplemental Figure 4 were analyzed via the CLC Sequence Viewer software. The gap open cost is set to 15.0, and extension cost is set to 1.0. The phylogenetic tree and PCC value in Figure 1 refer to the CAFRI-Rice (Hong et al., 2020) database.

**Molecular cloning.**

The guide RNA for CRISPR-Cas9 was designed by CRISPRdirect software (Naito et al., 2015). For single mutants, guide RNA was dimerized at 37˚C 1hours and ligated to pRGEB32-BsaI digested vector by T4 ligase. For multiple mutants, multiple guide RNA was ligated with tRNA by Golden gate (GG) reaction with T7 ligase and digested with FokI. The insert was ligated to the pRGEB32-BsaI vector by T4 ligase. For subcellular localization in pollen grain and tubes, *OsADFs* were expressed under their native promoter, 2000 upstream from the start codon. The monomeric red fluorescent protein (mRFP) was fused in the C-terminus of each OsADF1, OsADF6, and OsADF9. The promoter–coding sequence–mRFP was ligated to pGA3426-SpeI/HindIII digested vector by In-Fusion HD cloning enzyme (Takara). The plasmid vector was transformed into *O.sativa* L by *Agrobacterium*-mediated co-cultivation as previously reported (Lee et al., 1999). Primers used in this study are listed in Table S2.

**RNA extraction and real-time quantitative RT-PCR analysis**

Total RNA was extracted from 12–13 stage anthers using the RNeasy Plant Mini Kit (QIAGEN, Germany), and complementary DNA (cDNA) was synthesized with SuPrimeScript RT Premix (GeNetBio, Korea). In the four reported mutants, *rupo*, *osmtd2*, *gori,* and *osmadstri* (the triple mutant of *OsMADS62, OsMADS63*, and *OsMADS68*), three *OsADFs* (*OsADF1*, *OsADF6*, and *OsADF9)* expressions were analyzed via qRT-PCR. qRT-PCR was performed using a Rotor-Gene Q (QIAGEN, Germany) with the following cycle: 10 min at 95°C holding, 40 cycles with settings of 10s at 95°C – 15s at 60°C – 20s at 72°C, waiting for 90s of pre-melting and melting at 72°C to 95°C. The threshold of cycle (Ct) value was normalized by internal control (*OsUBI5; LOC_Os01g22490*) and gene expression value was calculated as described previously (Schmittgen & Livak, 2008). For qRT-PCR, nine tissues were selected: 7 days after germination (DAG) shoot; 7 DAG root; 60 DAG leaf; anthers at stages 5, 7, 9, 11, and 13 (mature anther); and developing seeds at one day after pollination.

**Pollen germination and histochemical assay**

The liquid PGM comprised 20% sucrose, 10% polyethylene glycol 4000, 3 mM Ca (NO_3_)_2_, 40 mg/L H_3_BO_3_, 10 mg/L thiamine. After anther dehiscence, fully mature pollen grains were collected in the PGM and incubated for 30 min at room temperature for *in vitro* germination. For starch staining, the tricellular stages pollen was stained with 1% I_2_-KI solution for 10 min at room temperature. For *in vivo* staining, the carpel was sampled 3–4 h after anther dehiscence and fixed with carnoy’s solution (with ethanol, acetic acid, and glycerol). The fixed samples were treated with 1 M NaOH and stained with 0.05% aniline blue for 1 h at room temperature. Germinated PT and stained PG were observed by the BX61 microscope (Olympus, Japan), and stained carpel was observed by the LSM800 (Carl Zeiss, Germany).

**Phenotypic analysis**

The fertility ratio refers to the seed set ratio per panicle, and the ratio of seed set for 20 panicles of each plant over 3 years was measured. Flowers were selected, including stages 12–13 anthers before dehiscence. For plant and panicle pictures, photos were taken 40–50 days after fertilization. PG and growing PT were observed by laser scanning microscope LSM510 (Carl Zeiss, Germany) in 558nm excitation filter settings.

**RNA sequencing and Bioinformatics analysis**

Protein sequences for the nine ADF genes were compiled through the RGAP database (http://rice.plantbiology.msu.edu/). The protein sequences were aligned by MEGA software and a phylogenetic tree was visualized by the neighbor-joining method with 1,000 times bootstraps (Tamura et al., 2021). The expression value of OsADFs was extracted at RNA-seq expression values registered in the CAFRI-Rice database, and the expression heatmap was visualized by MultiExperiment Viewer (MeV) software (Howe et al., 2010). For RNA sequencing analysis, the sequencing library was synthesized by the TruSeq Standard mRNA LT Sample Prep Kit (Illumina, United States), and sequencing was performed by the Illumina NovaSeq 6000 (Macrogen, Korea). The raw data of control and OsADF1/6/9 were mapped to the MSU7 version of the *Oryza sativa* genome and adapter trimming, mapping, and read counting analysis were followed as previously reported (Hong et al., 2022). Three biological replicates for the control and the mutant line were normalized with the DESeq2 packages in R studio. Differentially expressed genes (DEGs) were selected by the following standard: baseMean > 10; *p-value* < 0.05, *q-value* < 0.05, |log_2_Foldchange| > 2. Among the downregulated genes, genes with higher expression than OsUBI5 in pollen tissues were selected as the Downregulated (pollen) group. Gene Ontology (GO) analysis of downregulated genes was analyzed through agriGOv2 (http://systemsbiology.cau.edu.cn/agriGOv2/) web tool and visualized by the ggplot. GO features were sorted out by the following standards: *p-value* < 0.05; query number > 2; fold enrichment > 2.


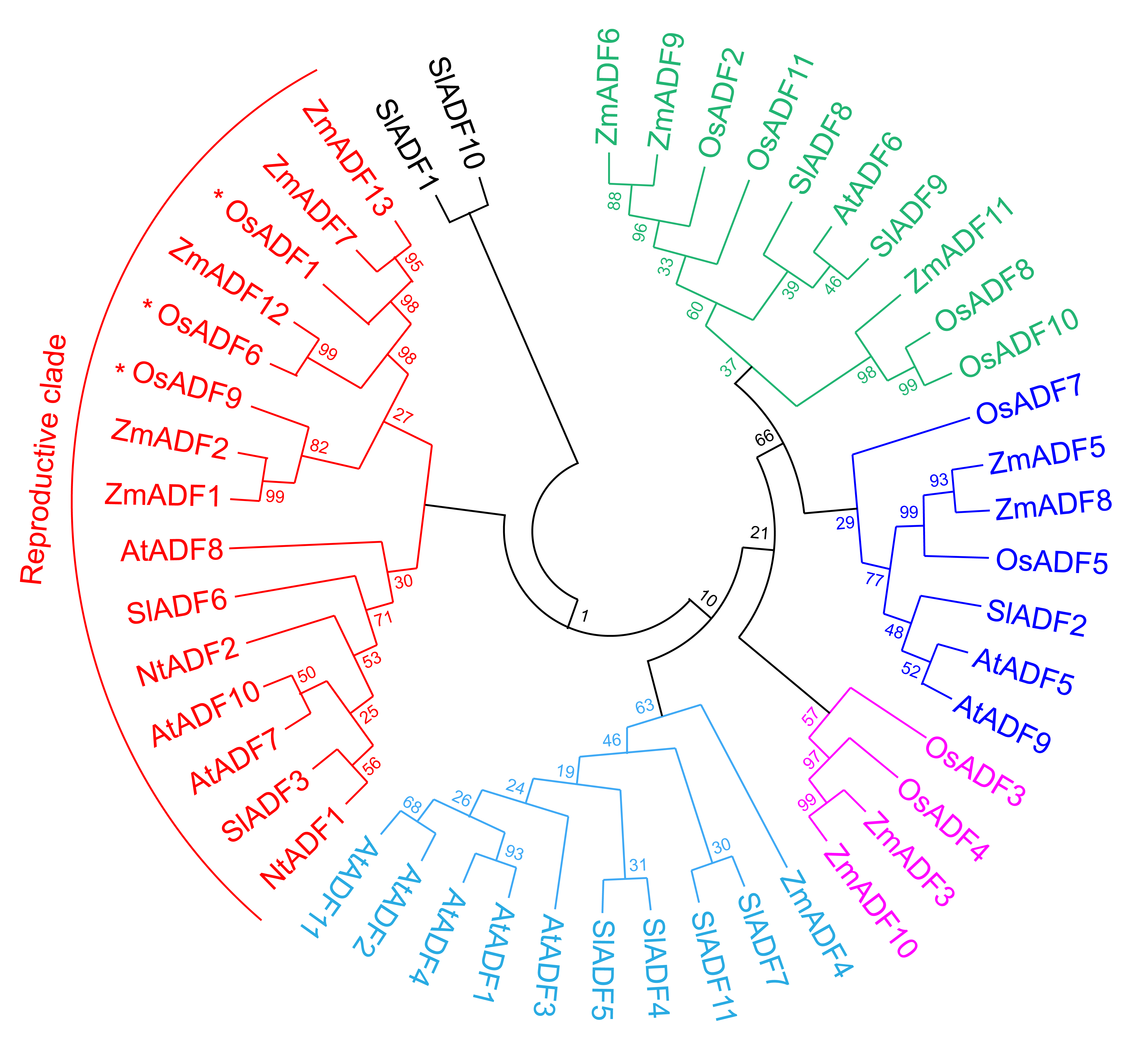


**Supplemental Figure S1.** Phylogenetic tree based on protein sequence similarities between OsADFs and ADFs in other species. Numbers in the tree indicate bootstrap values and colors represent each subgroup/subclade. Reproductive clades include genes that are highly expressed in anther and pollen, or genes whose functions are known in those tissues. Three rice genes; *OsADF1*, *OsADF6*, and *OsADF9*, also belong to the reproductive clade and are marked with asterisks. The locus number and protein sequence used for alignment and phylogenetic trees are listed in Supplementary Table 1.


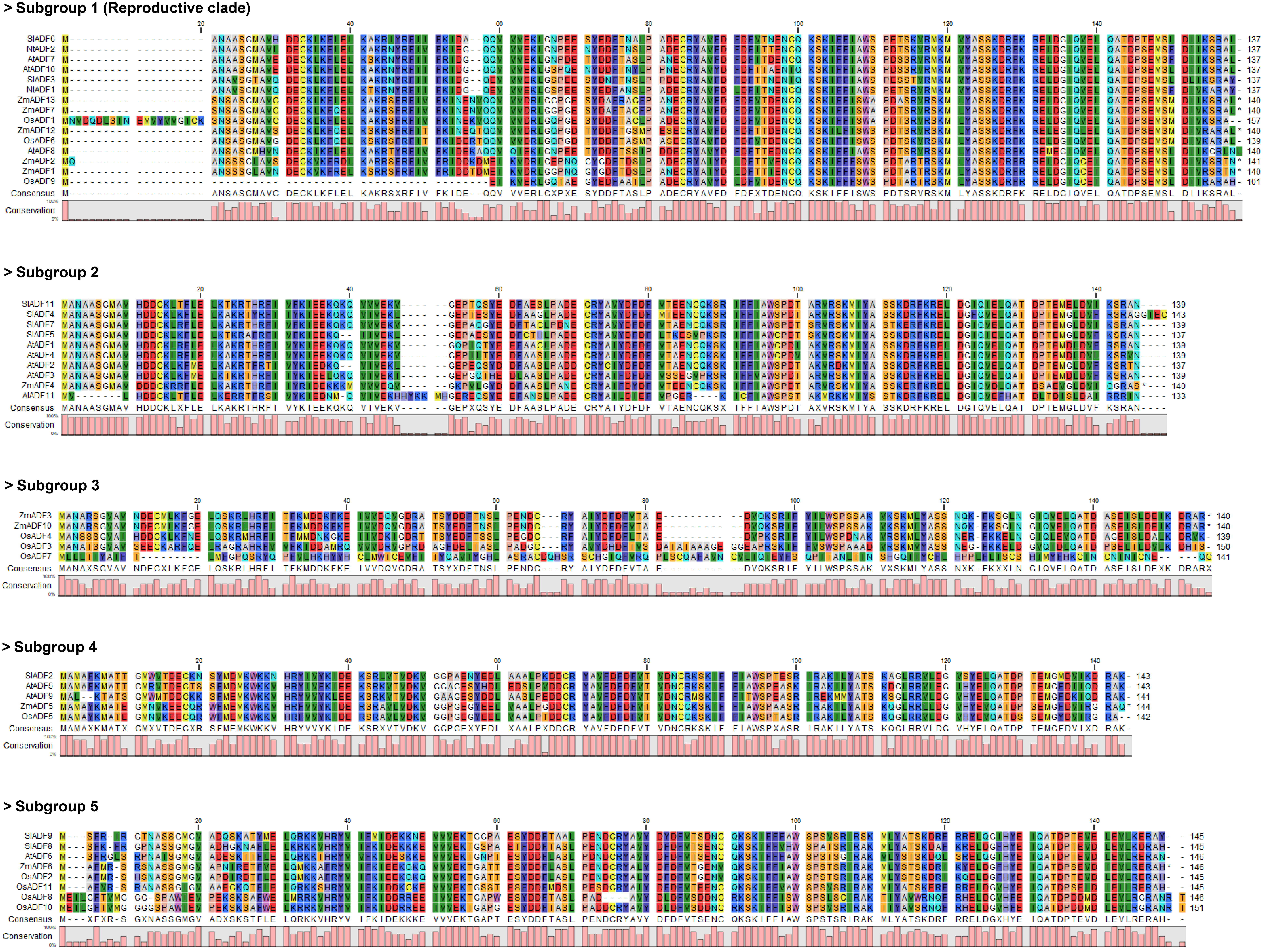


**Supplemental Figure S2.** Multiple sequence alignment of genes by subgroup based on phylogenetic tree. The sequences are aligned and represented by color for each amino acid, with consensus indicating the highest proportion of conserved bases and conservation means the frequency of consensus amino acids.


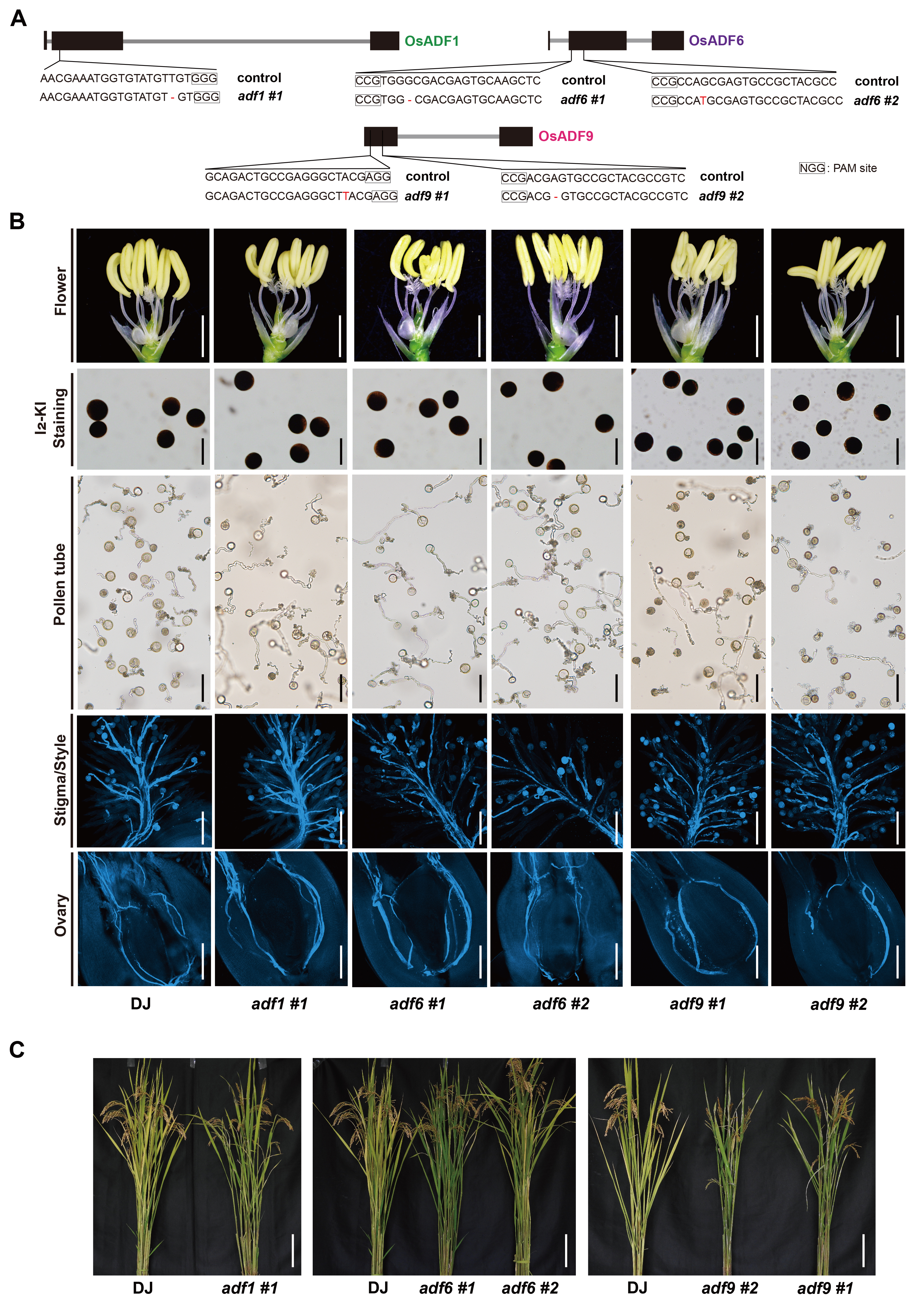


**Supplemental Figure S3.** gRNA target site and PG, PT, and plant pictures of single mutants. **A,** gRNA targeted sites and edited sequence of single mutants. **B,** Pictures of the flower, I_2_-KI stained mature PG, and PT for *in vitro* and *in vivo* germination analysis to compare DJ and series of single mutants. **C,** Pictures of the whole plant of DJ and single mutants. Bar, 20 cm (Plant), 2 mm (Flower), 50 μm (PG), 100 μm (*in vitro* PT), 200 μm (*in vivo* PT).


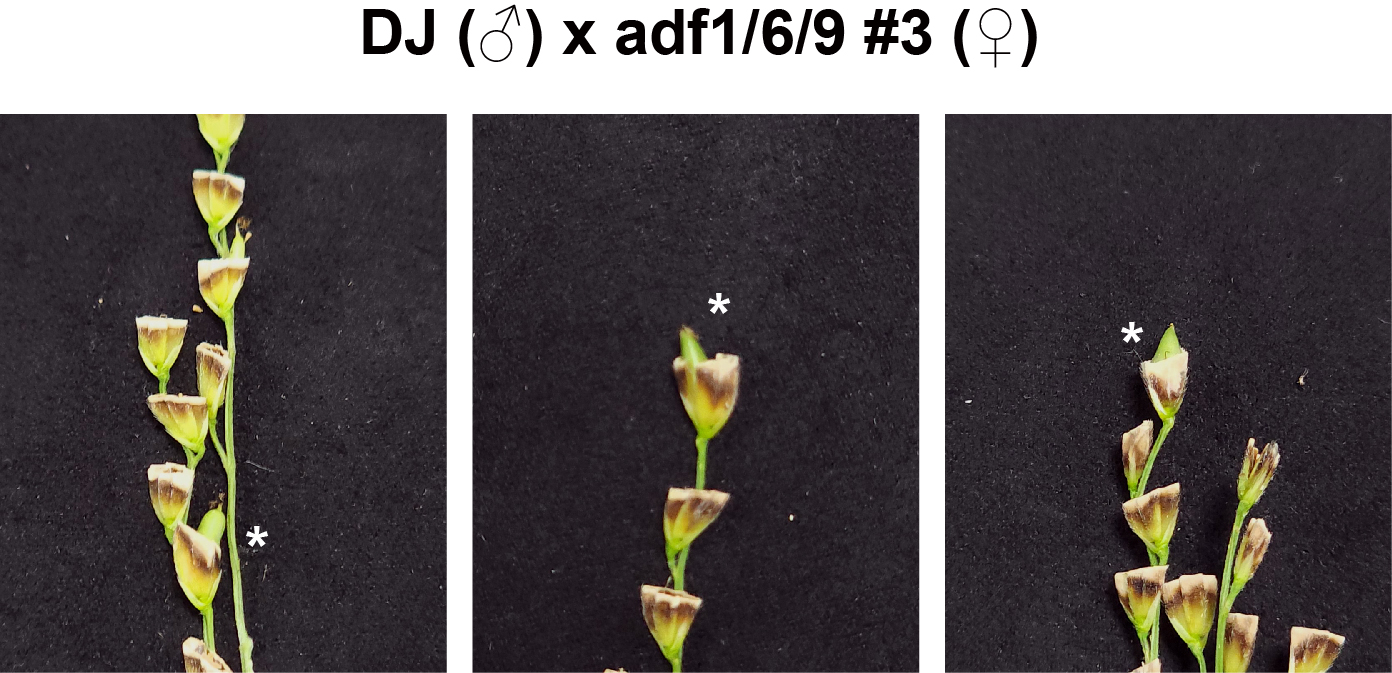


**Supplemental Figure S4.** Reciprocal crossing analysis between *adf1/6/9* ovaries and wild-type (DJ) pollen. White asterisks indicate the embryos after crossing.

**Supplemental Table S1.** Gene locus number and sequence used in phylogenetic analysis.

|  | **Gene Name** | **Locus Number** | **Protein sequence** |
| --- | --- | --- | --- |
| ***Oryza sativa*** | OsADF1 | Os02g44470 | MNVDQDLSINEMVYVVGICKSNSASGMAVCDECKLKFLELKAKRSFRFIVFKINEKVQQVVVDRLGQPGESYDDFTACLPADECRYAVFDFDFVTDENCQKSKIFFISWAPDTSRVRSKMLYASSKDRFKRELDGIQVELQATDPSEMSMDIVKSRA |
|  | OsADF2 | Os03g56790 | MAFMRSHSNASSGMGVAPDIRDTFLELQMKKAFRYVIFKIEEKQKQVVVEKTGATTESYDDFLASLPENDCRYALYDFDFVTGENVQKSKIFFIAWSPSTSRIRAKMLYSTSKDRIKQELDGFHYEIQATDPTEVDLEVLRERAH |
|  | OsADF3 | Os03g60580 | MANATSGVAVSEECKARFQELRAGRAHRFVVFKIDDAMRQVVVDRVGPRDAGFDELTASLPADGCRYAVYDHDFTVSDATATAAAGEGGEAPRSKIFFVSWSPAAADVRSKMVYASSNEGFKKELDGVQIDLQATDPSELTLDVLKDHTS |
|  | OsADF4 | Os03g60590 | MANSSSGVAIHDDCKLKFNELQSKRMHRFITFMMDNKGKEIIVDKIGDRTTSYEDFTSSLPEGDCRFAIYDFDFLTAEDVPKSRIFYILWSPDNAKVRSKMLYASSNERFKKELNGIQLEVQATDAGEISLDALKDRVK |
|  | OsADF5 | Os03g13950 | MAMAYKMATEGMNVKEECQRWFMEMKWKKVHRFVVYKIDERSRAVLVDKVGGPGEGYEELVAALPTDDCRYAVFDFDFVTVDNCQKSKIFFIAWSPTASRIRAKILYATSKQGLRRVLDGVHYEVQATDSSEMGYDVIRGRA |
|  | OsADF6 | Os04g46910 | MANSASGMAVGDECKLKFQELKSKRSFRFITFKIDERTQQVVVDRLGQPGDTYDDFTASMPASECRYAVFDFDFVTDENCQKSKIFFISWSPDTSKVRSKMLYASSKDRFKRELDGIQVELQATDPSEMSMDIVKARAL |
|  | OsADF7 | Os05g02250 | MLLLTIYAIFTLMFGPQSRYQPFVLHKHYYHCLMWTCEVFITYQAVIYGHLASRACDQHSRSCHGIQFVRQPLSCQAFAVNCVLIQIEYFSQPITANLTINSHGQIIYCFLHPPLFLISCSHIMYFHKCINCNINICNEQC |
|  | OsADF8 | Os07g20170 | MEILGFTVMGGGSPAWIEVPEKSKSAFWELMRRKVHRYVIFKIDDRREEIVVEKTGAPWESYDDFTASLPADAVYDLDFVSDDNCRKSKIFFISWSPSLSCIRAKTIYAVWRNQFRHELDGVHFEIQATDPDDMDLEVLRGRANRT |
|  | OsADF9 | Os07g30090 | MEIKVERLGQTAEGYEDFAATLPADECRYAVYDLDFVTDENCQKSKIFFFSWSPDTARTRSKMLYASSKDRFRRELDGIQCEIQATDPSEMSLDIIRARAH |
|  | OsADF10 | Os10g37670 | MEILGFTVMGGGGSPAWIEVPEKSKSAFWELKRRKVHRYVIFKIDDRREEIVVEKTGAPGESYDDFTASLPADDCRYAVYDLDFVSDDNCRKSKIFFISWSPSVSRIRAKTIYAVSRNQFRHELDGVHFEIQATDPDDMDLEVLRGRANRT |
|  | OsADF11 | Os12g43340 | MAFVRSRANASSGIGVAAECKQTFLELQRKKSHRYVIFKIDDKCKEVVVEKTGSSTESFDDFMDSLPESDCRYAIYDFDFVTEENCQKSKIFFVAWSPSVSRIRAKMLYATSKERFRRELDGVHYEIQATDPSELDIELLRERAH |
| ***Nicotiana tabacum*** | NtADF1 | LOC107783541 | MANAVSGMAVQDECKLKFLELKTKRNYRFIIFKIDGQEVVVEKLGSPEESYEDFANSLPADECRYAVFDLDFITNENCQKSKIFFIAWSPETSRVRMKMVYASSKDRFKRELDGIQVELQATDPSEMSFDIVKARAY |
|  | NtADF2 | LOC107783999 | MANAASGMAVLDECKLKFLELKAKRNYRFIVFKIEGQQVVVEKLGNPEENYDDFTNSLPADECRYAVFDFDFITTENCQKSKIFFIAWSPDTSKVRMKMVYASSKDRFKRELDGIQVELQATDPSEMSFDIIKSRAL |
| ***Arabidopsis thaliana*** | AtADF1 | At3g46010 | MANAASGMAVHDDCKLRFLELKAKRTHRFIVYKIEEKQKQVVVEKVGQPIQTYEEFAACLPADECRYAIYDFDFVTAENCQKSKIFFIAWCPDIAKVRSKMIYASSKDRFKRELDGIQVELQATDPTEMDLDVFRSRAN |
|  | AtADF2 | At3g46000 | MANAASGMAVHDDCKLKFMELKAKRTFRTIVYKIEDKQVIVEKLGEPEQSYDDFAASLPADDCRYCIYDFDFVTAENCQKSKIFFIAWSPDTAKVRDKMIYASSKDRFKRELDGIQVELQATDPTEMGLDVFKSRTN |
|  | AtADF3 | At5g59880 | MANAASGMAVHDDCKLKFMELKTKRTHRFIIYKIEELQKQVIVEKIGEPGQTHEDLAASLPADECRYAIFDFDFVSSEGVPRSRIFFVAWSPDTARVRSKMIYASSKDRFKRELDGIQVELQATDPTEMDLDVFKSRAN |
|  | AtADF4 | At5g59890 | MANAASGMAVHDDCKLRFLELKAKRTHRFIVYKIEEKQKQVIVEKVGEPILTYEDFAASLPADECRYAIYDFDFVTAENCQKSKIFFIAWCPDVAKVRSKMIYASSKDRFKRELDGIQVELQATDPTEMDLDVLKSRVN |
|  | AtADF5 | At2g16700 | MAMAFKMATTGMRVTDECTSSFMDMKWKKVHRYIVFKIEEKSRKVTVDKVGGAGESYHDLEDSLPVDDCRYAVFDFDFVTVDNCRKSKIFFIAWSPEASKIRAKILYATSKDGLRRVLEGIHYELQATDPTEMGFDIIQDRAK |
|  | AtADF6 | At2g31200 | MSFRGLSRPNAISGMGVADESKTTFLELQRKKTHRYVVFKIDESKKEVVVEKTGNPTESYDDFLASLPDNDCRYAVYDFDFVTSENCQKSKIFFFAWSPSTSGIRAKVLYSTSKDQLSRELQGIHYEIQATDPTEVDLEVLRERAN |
|  | AtADF7 | At4g25590 | MANAASGMAVEDECKLKFLELKSKRNYRFIIFRIDGQQVVVEKLGNPDETYDDFTASLPANECRYAVFDFDFITDENCQKSKIFFIAWSPDSSRVRMKMVYASSKDRFKRELDGIQVELQATDPSEMSFDIIKSRAL |
|  | AtADF8 | At4g00680 | MANSASGMHVNDECKIKFLELKAKRTYRFIVFKIDEKAQQVQIEKLGNPEETYDDFTSSIPDDECRYAVYDFDFTTEDNCQKSKIFFIAWSPDTSRVRSKMLYASSKDRFKREMEGIQVELQATDPSEMSLDIIKGRLNL |
|  | AtADF9 | At4g34970 | MALKTATSGMWMTDDCKKSFMEMKWKKVHRYVVYKLEEKSRKVTVDKVGAAGESYDDLAASLPEDDCRYAVFDFDYVTVDNCRMSKIFFITWSPEASRIREKMMYATSKSGLRRVLDGVHYELQATDPTEMGFDKIQDRAK |
|  | AtADF10 | At5g52360 | MANAASGMAVEDECKLKFLELKAKRNYRFIIFRIDGQQVVVEKLGSPQENYDDFTNYLPPNECRYAVYDFDFTTAENIQKSKIFFIAWSPDSSRVRMKMVYASSKDRFKRELDGIQVELQATDPSEMSLDIIKSRAL |
|  | AtADF11 | At1g01750 | MVLHDDCKLTFLELKERRTFRSIVYKIEDNMQVIVEKHHYKKMHGEREQSYEEFANSLPADECRYAILDIEFVPGERKICFIAWSPSTAKMRKKMIYSSTKDRFKRELDGIQVEFHATDLTDISLDAIRRRIN |
| ***Zea mays*** | ZmADF1 | GRMZM2G117603 | MANSSSGLAVNDECKVKFRELKSRRSFRFIVFRIDDTDMEIKVDRLGGPNQGYGDFTDSLPANECRYAIYDLDFTTIENCQKSKIFFFSWSPDTARTRSKMLYASSKDRFRRELDGIQCEIQATDPSEMSLDIVRSRTN* |
|  | ZmADF2 | GRMZM2G097122 | MQANSSSGLAVSDECKVKFRDLKARRSFRFIVFRIDDKDMEIKVDRLGEPNQGYGDFTDSLPADECRYAIYDLDFTTVENCQKSKIFFFSWSPDTARTRSKMLYASSKDRFRRELDGIQCEIQATDPSEMSLDIVKSRTN* |
|  | ZmADF3 | GRMZM2G060702 | MANARSGVAVNDECMLKFGELQSKRLHRFITFKMDDKFKEIVVDQVGDRATSYDDFTNSLPENDCRYAIYDFDFVTAEDVQKSRIFYILWSPSSAKVKSKMLYASSNQKFKSGLNGIQVELQATDASEISLDEIKDRAR* |
|  | ZmADF4 | GRMZM2G037140 | MANAASGMAVDDDCKRRFLELKAKRTHRFIIYRIDEKKKMVVVEQVGKPVLGYDDFAASLPANECRYAIFDYDFVTEENCQKSKIFFIAWSPDTARVRSKMIYASSKERFKRELDGIQVDLQATDSAEVGLDVIQGRAS* |
|  | ZmADF5 | GRMZM2G077942 | MAMAYKMATEGMNVKEECQRWFMEMKWKKVHRFVVYKIDERSRAVLVDKVGGPGEGYEELVAALPGDDCRYAVFDFDFVTVDNCQKSKIFFIAWSPAASRIRAKILYATSKQGLRRLLDGVHYEVQATDPSEMGFDVIRGRAQ* |
|  | ZmADF6 | GRMZM2G130678 | MAFMRSRSNASSGMGVAPNIRETFVELQMKKAFRYVIFKIEEKQKQVVVEKTGATTESYDDFLASLPENDCRYALYDFDFVTGENVQKSKIFFIAWSPSTSRIRAKMLYSTSKDRIKYELDGFHYEIQATDPSEVDIEVLRERAH* |
|  | ZmADF7 | GRMZM2G463471 | MSNSASGMAVCDECKLKFQELKAKRSFRFIVFKINENVQQVVVDRLGGPGESYDAFTACFPANECRYAVFDFDFVTDENCQKSKIFFISWAPDTSRVRSKMLYASSKDRFKRELDGIQVELQATDPSEMSMDIVKSRAL* |
|  | ZmADF8 | GRMZM2G147775 | MRDRIRRQRIPRFRRQGVHPIRDDPQATAMAMAYKMATEGMDVKEECQRWFMEMKWKKVHRFVVYRIDERSRAVLVDRVGGPGEGYEELVAALPGDDCRYAVFDFDFVSVDNCQKSKIFFIAWSPAASRIRAKILYATSKQGLRRLLDGVHYEVQATDPSEMGFDVIRGRAQ* |
|  | ZmADF9 | GRMZM2G108807 | MGVALKIRETFVELQMKKAFRYVIFKIEEKQKQVVVEKTGATTKNYDDFLASLLENDCRYALYDFDFVTRENVQKSKIFFIAWSPSTSRIRAKMLYSTSKDRIKYELDGFHYEIQATDPSEVDIEVLRERAH* |
|  | ZmADF10 | GRMZM2G002825 | MANARSGVAVNDECMLKFGELQSKRLHRFLTFKMDDKFKEIVVDQVGDRATSYEDFTNSLPENDCRYAIYDFDFVTAEDVQKSRIFYILWSPSSAKVKSKMLYASSNQKFKSGLNGIQVELQATDASEISLDEIKDRAR* |
|  | ZmADF11 | GRMZM2G064875 | MEVLGFAAMGGGSPAWIDVPERSKSAFMELKRRKVHRYVIFKIDDSREEVVVDKIGAPGESYDDFTASLPTDDCRYAVYDLDFVSDDNCRKSKIFFISWSPSDSRIRAKTIYAVSRNQFRHELDGVHFEIQATDPDDMNLEVLRGRANRT* |
|  | ZmADF12 | GRMZM2G071327 | MANSASGMAVSDECKLKFQELKSKRSFRFITFKINEQTQQVVVDRLGQPGDTYDDFTGSMPESECRYAVFDFDFTTDENCQKSKILFISWSPDTSRVRSKMLYASSKDRFKRELEGIQLELQATDPSEMSMDIVRARAL* |
|  | ZmADF13 | GRMZM2G015127 | MSNSASGMAVCDECKLKFLELKAKRSFRFIVFKINENVQQVVVDRLGGPGESYDAFRACFPANECRYAVFDFDFVTDENCQKSKIFFISWAPDASRVRSKMLYASSKDRFKRELDGIQVELQATEPSEMSMDIIKSRAL* |
| ***Solanum lycopersicum*** | SlADF1 | Solyc01g094400 | MANSASGIAVSDECKLKFMELKAKRNHRYIVFKIDGQQVVVEKVGGQAETHDDLANSLPPNECRYAVFDYDFTTNENVQKSKIFFIAWSPETARVRSKMLYASSKDRFRREFDGVQVELQATDPSEMSLDTFICRAL |
|  | SlADF2 | Solyc01g111380 | MAMAFKMATTGMWVTDECKNSYMDMKWKKNHRYIVYKIDEKSRLVTVDKVGGPAENYEDLAAALPKDDCRYAVFDFDFVTVDNCRKSKIFFIAWSPTESRIRAKILYATSKAGLRRVLDGVSYELQATDPTEMGMDVIKDRAK |
|  | SlADF3 | Solyc03g025750 | MANAVSGTAVQDECKLKFLELKAKRNYRFIIFKIDGQEVVVEKLGSPEESYDNFTNSLPPDECRYAVFDFDFTTNENIQKSKIFFIAWSPESSTVRMKMVYASSKDRFKRELDGIQVELQATEPSEMSLDLIKSRAY |
|  | SlADF4 | Solyc04g011370 | MANAASGMAVHDDCKLKFLELKAKRTYRFIIYKIEEKQKQVIVEKLGEPTESYEDFAAGLPADECRYAVYDFDFMTEENCQKSRIFFIAWSPDTARVRSKMIYASSKDRFKRELDGFQVELQATDPTEMGLDVFRSRAGGIEC |
|  | SlADF5 | Solyc06g005360 | MANAASGMAVHDDCKLKFLELKTKRAFRFIVFKIEEKQIIVEKLGEPAESYEDFCTHLPADECRYAVYDFDFLTKESVPKSRIFFIAWCPDTSKVRSKMIYASSKDRFKRELDGIQIELQATDPTEMGLDVFKSRAN |
|  | SlADF6 | Solyc06g035980 | MANAASGMAVHDDCKLKFLELKAKRIYRFIIFKIDAQQVVVEKLGNPEESYEDFTNALPADECRYAVFDFDFVTNENCQKSKIFFIAWSPETSKVRMKMVYASSKDRFKREIDGIQVELQATDPTEMSLDIIKSRAL |
|  | SlADF7 | Solyc09g010440 | MANAASGMAVHDDCKLRFLELKAKRTHRFIVFKIEEKQKQVVVEKVGEPAQGYEDFTACLPDNECRYAVYDFDFVTAENCQKSRIFFIAWSPDTSRVRSKMIYASTKERFKRELDGIQVELQATDPTEMGLDVIKSRAN |
|  | SlADF8 | Solyc09g072590 | MSFKFRGPNASSGMGVADHGKNAFLELKRKKVHRYVIFKIDEKKKEVVVEKTGSPAETFDDFTASLPENDCRYAVYDFDFVTSENCQKSKIFFVHWSPATSRIRAKMLYATSKDAFKRELDGFHYEIQATDPTEVDLEVLKDRAH |
|  | SlADF9 | Solyc09g090110 | MSFRIRGTNASSGMGVADQSKATYMELQRKKVHRYVIFMIDEKKNEVVVEKTGGPAESYDDFTAALPENDCRYAVYDYDFVTSDNCQKSKIFFFAWSPSVSRIRSKMLYATSKDRFRRELQGIHYEIQATDPTEVELEVLKERAY |
|  | SlADF10 | Solyc10g017550 | MANSASGIAVSDECKLRFLELKAKRNHRYLVFKIDESVQQVVVEKVGGQTETHDDFANSMPPSECRYAVFDYDWTTDENVQKSRIFFVAWSPETARVRSKMLYASSKDRFRRELDGVQVELQATDPSEMSLDTFVGRIH |
|  | SlADF11 | Solyc10g084660 | MANAASGMAVHDDCKLTFLELKTKRTHRFIVFKIEEKQKQVVVEKVGEPTQSYEDFAESLPADECRYAVYDFDFVTEENCQKSRIFFIAWSPDTARVRSKMIYASSKDRFKRELDGIQIELQATDPTEMELDVIKSRAN |

**Supplemental Table S3.** Primer list used in this study.

|  | **Primer Name** | **Primer Sequence (5'-3')** |
| --- | --- | --- |
| **qRT-PCR** | OsADF1-qRT-F | AGCAGATGAGTGCCGCTACG |
|  | OsADF1-qRT-R | GATCCTTGGAGCTAGCATAC |
|  | OsADF6-qRT-F | ACACCTACGACGACTTCACC |
|  | OsADF6-qRT-R | TCCTCACCTTCGACGTGTCC |
|  | OsADF9-qRT-F | AGACTGCCGAGGGCTACGAG |
|  | OsADF9-qRT-R | AATCTCGCACTGGATTCCGT |
|  | OsUBI5-qRT-F | ATGCGTAGGTTGGACGTCAT |
|  | OsUBI5-qRT-R | AGAAACTGATCTCCCTGCCG |
| **Cloning** | OsADF1-mCas-F | TAGGTCTCCGGTGTATGTTGTGTTTTAGAGCTAGAA |
|  | OsADF1-mCas-R | CGGGTCTCACACCATTTCGTTTGCACCAGCCGGG |
|  | OsADF6-mCas-F | TAGGTCTCCAACTCAGCGTCG GTTTTAGAGCTAGAA |
|  | OsADF6-mCas-R | CGGGTCTCAAGTTCGCCTGCATGCACCAGCCGGG |
|  | OsADF9-mCas-F | TAGGTCTCCCCGAGGGCTACGGTTTTAGAGCTAGAA |
|  | OsADF9-mCas-R | CGGGTCTCATCGGCAGTCTGCTGCACCAGCCGGG |
|  | OsADF1-sCas-target1-F | GGCAAACGAAATGGTGTATGTTGT |
|  | OsADF1-sCas-target1-R | AAACACAACATACACCATTTCGTT |
|  | OsADF6-sCas-target1-F | GGCAGAGCTTGCACTCGTCGCCCA |
|  | OsADF6-sCas-target1-R | AAACTGGGCGACGAGTGCAAGCTC |
|  | OsADF6-sCas-target2-F | GGCAGGCGTAGCGGCACTCGCTGG |
|  | OsADF6-sCas-target2-R | AAACCCAGCGAGTGCCGCTACGCC |
|  | OsADF9-sCas-target1-F | GGCAGCAGACTGCCGAGGGCTACG |
|  | OsADF9-sCas-target1-R | AAACCGTAGCCCTCGGCAGTCTGC |
|  | OsADF9-sCas-target2-F | GGCAGACGGCGTAGCGGCACTCGT |
|  | OsADF9-sCas-target2-R | AAACACGAGTGCCGCTACGCCGTC |
|  | OsADF1-mRFP-promoter-F | TACCTATTTCCGCCCGGATCCGGGATCGACCGTGCCACGGT |
|  | OsADF1-mRFP-promoter-R | AGATCTTGATCAACATTCATGGTTGATGATCCTTTTTTTT |
|  | OsADF1-mRFP-CDS-F | AAAAAAAAGGATCATCAACCATGAATGTTGATCAAGATCT |
|  | OsADF1-mRFP-CDS-R | CGTTGTACACGTACGAAGCTTGAGGGCTCGCGACTTGACGA |
|  | OsADF6-mRFP-promoter-F | TACCTATTTCCGCCCGGATCCCCATTGAAAATGGTAAATAA |
|  | OsADF6-mRFP-promoter-R | CCCGACGCTGAGTTCGCCATGGTTGATAGGTTTCGATCTC |
|  | OsADF6-mRFP-CDS-F | GAGATCGAAACCTATCAACCATGGCGAACTCAGCGTCGGG |
|  | OsADF6-mRFP-CDS-R | ACGCGTTGTACACGTACGAAGCTTGAGGGCTCTCGCTTTGACGA |
|  | OsADF9-mRFP-promoter-F | TACCTATTTCCGCCCGGATCCATCATATTCGATCCTTATTT |
|  | OsADF9-mRFP-promoter-R | CTCTCCACCTTGATCTCCAT GGCCTTGTCGTCGATCTTGA |
|  | OsADF9-mRFP-CDS-F | TCAAGATCGACGACAAGGCCATGGAGATCAAGGTGGAGAG |
|  | OsADF9-mRFP-CDS-R | CGTTGTACACGTACGAAGCTTGTGAGCTCTGGCTCTGATGA |

**References**

**Hong WJ, Kim EJ, Yoon J, Silva J, Moon S, Min CW, Cho LH, Kim ST, Park SK, Kim YJ.** A myosin XI adaptor, TAPE, is essential for pollen tube elongation in rice. Plant Physiol. 2022:190(1): 562-575. https://doi.org/10.1093/plphys/kiac299

**Hong W, Kim Y, Kim E, Kumar Nalini Chandran A, Moon S, Gho Y, Yoou M, Kim ST, Jung K.** CAFRI‐Rice: CRISPR applicable functional redundancy inspector to accelerate functional genomics in rice. Plant J. 2020:104(2): 532-545. https://doi.org/10.1111/tpj.14926

**Howe, E., Holton, K., Nair, S., Schlauch, D., Sinha, R., & Quackenbush, J.** Mev: multiexperiment viewer. Biomedical Informatics for Cancer Research. 2010:267-277.

**Kawahara, Y., de la Bastide, M., Hamilton J. P., Kanamori, H., McCombie, W. R., Ouyang, S., Schwartz, D. C., Tanaka, T., Wu, J., Zhou, S., Childs, K. L., Davidson, R. M., Lin, H., Quesada-Ocampo, L., Vaillancourt, B., Sakai, H., Lee, S. S., Kim, J., Numa, H., Itoh, T., Buell, C. R., Matsumoto, T.** Improvement of the Oryza sativa Nippon bare reference genome using next generation sequence and optical map data. Rice*.* 2013:**6**:4. https://doi.org/10.1186/1939-8433-6-4

**Kumar S, Stecher G, Li M, Knyaz C, and Tamura K.** MEGA X: Molecular Evolutionary Genetics Analysis across Computing Platforms. Mol. Biol. Evol. 2018:35(6):1547-1549. https://doi.org/10.1093/molbev/msy096

**Lee, S., Jeon, J., Jung, K. and An, G.** Binary vectors for efficient transformation of rice. J. Plant Biol. 1999:42: 310-316. https://doi.org/10.1007/BF03030346

**Naito, Y., Hino, K., Bono, H., & Ui-Tei, K.** CRISPRdirect: software for designing CRISPR/Cas guide RNA with reduced off-target sites. Bioinformatics, 2015:31(7):1120-1123. 10.1093/bioinformatics/btu743.

**Schmittgen TD, Livak KJ. 2008.** Analyzing real-time PCR data by the comparative CT method. Nature protocols. 2008:3:1101-1108. https://doi.org/10.1038/nprot.2008.73

**Tamura K, Stecher G, Kumar S. 2021.** MEGA11: Molecular Evolutionary Genetics Analysis Version 11. Mol. Biol. Evol. 2021:38(7): 3022-3027. https://doi.org/10.1093/molbev/msab120
